# Synthesis and Characterization of Chitosan-Coated Near-Infrared (NIR) Layered Double Hydroxide-Indocyanine Green Nanocomposites for Potential Applications in Photodynamic Therapy

**DOI:** 10.3390/ijms160920943

**Published:** 2015-09-01

**Authors:** Pei-Ru Wei, Yaswanth Kuthati, Ranjith Kumar Kankala, Chia-Hung Lee

**Affiliations:** Department of Life Science and Institute of Biotechnology, National Dong Hwa University, Hualien 974, Taiwan; E-Mails: wei62879@gmail.com (P.-R.W.); yaswanthk1987@gmail.com (Y.K.); ranjithkankala@yahoo.com (R.K.K.)

**Keywords:** chitosan, indocyanine green, layered double hydroxide, nanoparticles, photodynamic therapy

## Abstract

We designed a study for photodynamic therapy (PDT) using chitosan coated Mg–Al layered double hydroxide (LDH) nanoparticles as the delivery system. A Food and Drug Administration (FDA) approved near-infrared (NIR) fluorescent dye, indocyanine green (ICG) with photoactive properties was intercalated into amine modified LDH interlayers by ion-exchange. The efficient positively charged polymer (chitosan (CS)) coating was achieved by the cross linkage using surface amine groups modified on the LDH nanoparticle surface with glutaraldehyde as a spacer. The unique hybridization of organic-inorganic nanocomposites rendered more effective and successful photodynamic therapy due to the photosensitizer stabilization in the interlayer of LDH, which prevents the leaching and metabolization of the photosensitizer in the physiological conditions. The results indicated that the polymer coating and the number of polymer coats have a significant impact on the photo-toxicity of the nano-composites. The double layer chitosan coated LDH–NH_2_–ICG nanoparticles exhibited enhanced photo therapeutic effect compared with uncoated LDH–NH_2_–ICG and single layer chitosan-coated LDH–NH_2_–ICG due to the enhanced protection to photosensitizers against photo and thermal degradations. This new class of organic-inorganic hybrid nanocomposites can potentially serve as a platform for future non-invasive cancer diagnosis and therapy.

## 1. Introduction

Highly beneficial qualities of photodynamic therapy (PDT) include its selective and minimally invasive nature with preferential localization of photosensitizer at the desired site along with concomitant photoactivation to restrict damage. PDT has been gaining significant attention as a very good alternative to surgery and drug resistance. Recently, PDT was demonstrated to be very effective in treating various malignancies [1,2,3,4,5,6,7]. However, currently available photosensitizers are hydrophobic in nature and easily aggregate during the delivery process, which consequently reduces the photodynamic action by encumbering their systemic administration [8,9,10]. In addition, the thermal degradation of photosensitizers upon the influence of heat produced by light irradiation is problematic for drug administration *in vivo*. The selectivity of a photosensitizer is very important, because light penetration during PDT to the targeted organs is indeed very poor. Further, side-effects associated with prolonged PDT exposure restricts their usage in clinical settings [10,11,12]. There is a need to design a nanocarrier that can transport photosensitizers in an efficient way to the targeted site with rapid clearance rates. Many polymer-based nanoparticle drug delivery systems that were synthesized by using nanotechnology have entered clinics [13,14,15] and currently a great number are in development at preclinical stages [16,17,18]. One of the most interesting designs of nanotechnology is the layered nanostructures developed by Swedish scientists; the layered double hydroxide (LDH) materials are well-known for their peculiar characteristics like controllable size and morphology, better biocompatibility, highly chemical stability, ion-exchanged properties for drug loading and release [7]. These properties for the LDH vehicle can be designed as smart delivery systems to transport various drug molecules, tracking probes, antibodies, enzymes and therapeutic genes for biological applications [7,19].

To design a drug delivery system that can carry and release the drug at the targeted site is an extremely critical area in the field of nanopharmaceutics [20]. Recently there has been remarkable development in the field to design controlled drug release by using LDH nanomaterials [21,22,23,24,25,26]. The use of LDH as a drug delivery system has been shown to increase biocompatibility, bioavailability [26] and solubility of hydrophobic drugs [20,27,28,29,30]. In current studies, the use of nanoparticles for drug delivery is challenging due to negatively charged frameworks that may restrict the effective delivery of the drug vehicle to enter cells. LDH nanoparticles have a positively charged framework, which is easy for uptake through adsorption by the cells possessing negatively charged cell membranes without additional post-modification. Choy and his group reported a very remarkabe neutrally or positively charged LDH nanoparticle design that are highly efficient in attaching to most anionic molecules facilitating an easy internalization through the cell membranes [31,32]. The nanoparticles are taken up into cells through clathrin-mediated endocytosis [33,34,35]. In addition, these nanoparticles were also demonstrated to efficiently deliver biological molecules [36,37,38,39,40]. Recently, hybrid nanocomposites showed the advantages of several nanoparticles in a single drug formulation with good flexibility to enhance properties such as drug dissolution [41], bioavailability [42,43], physical stability [44], and *in vivo* imaging [45,46]. Naked forms of LDH are not suitable for imaging due to their biodegradability in acidic pH, which leads to leaching and precipitation of the loading dyes. To overcome this, we have synthesized the chitosan coated near-infrared LDH nanoparticles for *in vivo* optical imaging [19]. The coating of nanoparticles with positively charged chitosan, *i.e.*, a natural polysaccharide derived from chitin (poly-β-(1→4)-*N*-acetyl-d-glucosamine) imparts beneficial qualities in a wide range of industries such as food, agricultural, cosmetic and pharmaceutical [47,48,49]. The chitosan structure with a cationic amine group is used to develop various nano-formulations for drug delivery and reveals a possible pathway for chitosan use in smart molecular devices responsive to external pH-stimuli to aid in designing various pH-responsive drug delivery systems [50].

To date, the use of LDH materials for PDT applications is barely studied. Very recently, Wang *et al.* provided a nanohybrid of co-loading Pt(IV) prodrugs and photosensitizers (Chlorin e6) into LDH for synergistic killing effects in cisplatin-resistant cancer cells [51]. A pH-responsive release of hydrophilic zinc(II) phthalocyanine based on electrostatic interaction with cationic LDH to generate high photocytotoxicity against HepG2 cells was reported by Li *et al.* [52]. Stefanakis *et al.* reported the synthesis of amino-modified Gd_2_(OH)_5_NO_3_ nanosheets with a photosensitiser (rose bengal). This nanocomposite makes photodynamic therapy possible [53]. Very interestingly, Liang *et al.* incorporated zinc phthalocyanines (ZnPc) into the gallery of LDHs by co-precipitation. The supramolecular photosensitizers showed a high photocytotoxicity, high stability, good biocompatibility as well as low cytotoxicity in comparison with pristine ZnPc [54]. In addition, many studies also demonstrated the incorporation of a porphyrin-based photosensitizer in the LDH host can enhance the stability of photosensitizers against the photobleaching and quenching aggregation effects [55,56,57,58].

**Scheme 1:**
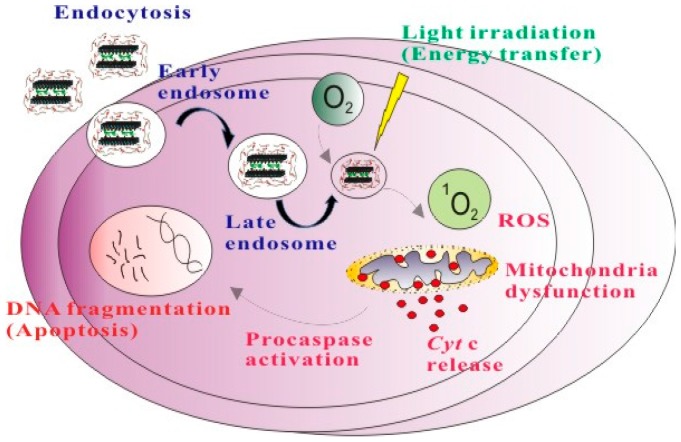
Representation of the cellular uptake of chitosan coated layered double hydroxide (LDH)–NH_2_–indocyanine green (ICG) nanohybrids and resultant apoptosis with photodynamic therapy (PDT).

Herein, we designed a chitosan-based delivery system for PDT based on chitosan-coated LDH nanoparticles loaded with a Food and Drug Administration (FDA)-approved near-infrared (NIR) fluorescent dye, indocyanine green (ICG) with photoactive properties. The surface coating of LDH with chitosan layers may improve the biological properties such as biocompatibility, immune responses, cell internalization and therapeutic effects [59]. In addition, the chitosan coating over LDH nanoparticles may enhance the photosensitizer excitation efficiency, which is highly beneficial for the application of LDHs in biologicals. The efficient positively charged polymer (chitosan) coating was achieved by the cross linkage using surface amine groups modified on the LDH nanoparticles surface with glutaraldehyde as a spacer and the photodynamic efficacy of ICG was demonstrated (Scheme 1).

## 2. Results and Discussion

ICG is an FDA-approved NIR fluorescent dye which has very attractive features like low cytotoxicity and high absorption at wavelength of 800–805 nm, which is a relatively transparent window for the biological tissues and solution. Further, the excitation of dye using NIR light has the advantage of increasing penetration depths in tissues. However, the easy biodegradation and the photo instability of ICG may limit the biological applications. This has been overcome by encapsulating ICG dye in a biocompatible matrix of layered double hydroxide nanoparticles and further coating them with a natural polymer chitosan.

### 2.1. Physical Characterization of Chitosan-Coated Layered Double Hydroxide (LDH)–NH_2_–Indocyanine Green (ICG) Matrix

**Figure 1:**
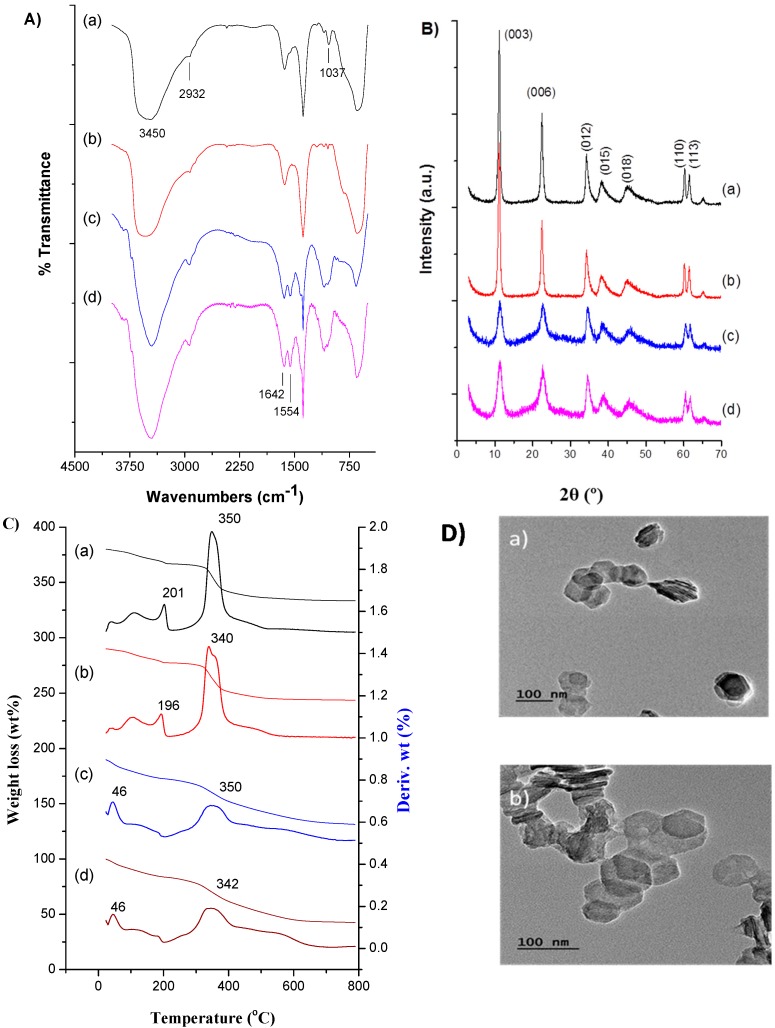
(**A**) FT-IR spectra; (**B**) PXRD patterns and (**C**) TGA curves of (a) LDHs–NH_2_–ICG (no irradiation); (b) LDHs–NH_2_–ICG (light irradiation); (c) LDHs–NH_2_–ICG–CS-1 (no irradiation) and (d) LDHs–NH_2_–ICG–CS-1 (light irradiation). light exposure, *i.e.*, after irradiation for 5 min; (**D**) TEM images of the characteristic hexagonal shape of LDHs–NH_2_–ICG–CS-1, before (a) and after (b) light exposure.

Physical characterization of this formulation includes chitosan-coated LDH nanocarriers and previous examples were reported [19]. The focus of this work is on the PDT effect, herewith, we analyzed the photo dynamic changes of the same formulation and the results of characterization (FT-IR, XRD, TGA and TEM) are discussed. The FT-IR spectrum of the LDH–NH_2_–ICG sample is represented in Figure 1A(a) and the PDT effect on the particle was further evaluated by exposure to the light at a prescribed time interval for 5 min (Figure 1A(b)) and resulted in no change in the spectrum, which demonstrates that the light effect is insignificant for the particle stability. The band at 1638 cm^−1^ is presumably caused by H_2_O deformation and at 3450 cm^−1^ is due to the O–H stretch of the absorbed H_2_O molecules and the hydroxyl groups in the LDH structures (Figure 1A(a)). The absorption peak at 672 cm^−1^ was attributed to the vibration of (M–O–M) (M = Mg or Al). A series of vibrations at 1556 and 2932 cm^−1^ attributed to N–H (primary amine) bending vibration and C–H stretching confirms the surface amination. The peak at 1382 cm^−1^ is reduced to some extent, which indicates the exchange of nitrate ions in the interlayer of LDH. The peak at 1037 cm^−1^ (Figure 1A(a)) is due to the traces of ethanol (primary alcohol) in the sample used to prevent contamination during sample storage. This also suggests the chitosan-coated LDH sample (Figure 1A(c)) has no change in functional groups and was preserved well after exposure to the light (Figure 1A(d)). The peaks at 1554 and 1642 cm^−1^ are due to N–H absorption and –C=N vibrations from the extra contribution of the chitosan molecules with a glutaraldehyde linker.

Figure 1B, illustrates the typical powder X-ray diffraction (PXRD) patterns of various nanoparticles with a structural featured characteristics shown by a hydrotalcite. The three distinct and intense peaks of basal reflections (2θ) at 11.0°, 23.3° and 34.9° (Figure 1B(a)) corresponds to planes d(003), d(006) and d(012), with sharp and symmetric peaks characteristic of LDHs structure. In addition, the characteristic peaks at planes d(110) and d(113) of basal reflection 60.8° and 62.2° indicate a typical layered hexagonal structural arrangement, that has created an opportunity to exchange anions between layers in order to adjust in the interlayer space. Because of very low loading, no significant change in d-spacing was observed after loading ICG (Figure 1B(b)) [19]. The light has no effect on the crystallinity of LDH–NH_2_–ICG and the spectrum was almost the same. Similarly, the chitosan-coated sample was subjected to the light exposure for the prescribed time and changes observed were insignificant (Figure 1B(c,d)). Nevertheless, crystallinity was slightly disturbed during the external modifications. The same results were also reported for the multiple coatings of polymer on the surface of the LDH leading to disorder of stacked metal hydroxide layers due to exfoliation of the LDH and decrease in the intensity of the peak [60]. ICG loading in the LDH was confirmed by comparing the ICG loaded LDH with the physical mixture of ICG (free) and LDH–NH_2_ using FT-IR (Figure S1) and TGA (Figure S2) analysis. The weight loss curves of TGA in all the respective samples of LDH resulted in no changes after exposure to the light (Figure 1C). LDH samples exhibited different stages of weight loss; the first is attributed to water loss (3–5 wt %) from the surface due to physical absorption and the interlayer space of LDH nanoparticles below 100 °C. The next event of weight loss after 200 °C was due to the degradation of organic linkers (amine) to LDH as well as counter ions (NO_3_^−^) and the dehydroxylation of the layered structure, the weight loss is around 28%–30% in LDH–NH_2_–ICG and higher (~40%) in case of chitosan coated nanohybrids (Figure 1C). However, the final stage of the degradation shifted right and extended to 450 °C with ~30% weight loss (200–450 °C) (Figure S2c) and compared with the physical mix of LDH–NH_2_ and ICG (Figure S2d), demonstrating that the ICG and LDH were packed together and shrouded within the layers. Since the ICG-loaded nanovehicles are degraded at higher temperatures, the enhanced thermal stability supports the strength of interactions between the ICG molecules in the interlayer gallery as well. This concomitantly proves the light has no effect on the stability of the LDH and that stability improved after chitosan coating on the surface of LDH–NH_2_ nanohybrids. The synthesized nanoconjugates are well shaped hexagonally as usual, with the lateral dimension in 100 nm both in the presence (Figure 1D(b)) and absence of light (Figure 1D(a)) with slight aggregation. This suggests that the morphological features of the layered double hydroxide nanoparticles after coating with a layer of chitosan are well preserved. In addition, NIR light irradiation has no significant effect on the structure of LDH.

### 2.2. Photostability of ICG in LDH–Chitosan Matrix

**Figure 2:**
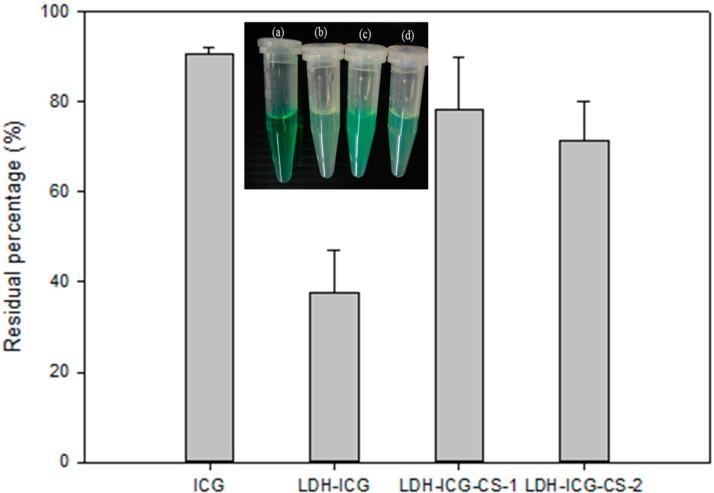
Photostability experiments of absorption residual for (**a**) free ICG; (**b**) LDH–NH_2_–ICG; (**c**) LDH–NH_2_–ICG–CS-1 and (**d**) LDH–NH_2_–ICG–CS-2, aqueous suspensions after exposure with light irradiation only for 3 min and after three hours; the absorbance of samples was read using a UV–Visible spectrophotometer.

The encapsulation of organic molecules into the interlayer of LDHs can significantly increase their stability. In the case of the ICG fluorescent dye, photobleaching remains a major hurdle, which may degrade the chromophore of dyes. To date, no quantitative studies have been reported to compare the biological stability of free ICG and ICG incorporated LDH interlayers. Accordingly, the stability of ICG upon irradiation of light (NIR-765 nm) was investigated using a free ICG base (aqueous solution), ICG loaded LDH particles with single and double chitosan coatings (Figure 2).

The absorption and fluorescence spectrum of ICG is in the near infrared region with an intense band at 765 nm in water. The free base ICG (Figure 2a) has shown photo stability as monitored by the maintenance of optical density of the Soret band at 765 nm reaching 90% after 180 min. On the other hand, the LDH–NH_2_–ICG sample exposed at 765 nm showed loss of absorbance for the Soret band (Figure 2b) and a light colour change was observed. The loss of absorbance reached around 70% after irradiation. Therefore, a highly electrostatic attraction between the positively charged LDH surfaces and the negatively charged ICG molecules may cause decreased absorbance due to a local aggregation of ICG molecules at the LDH surfaces. Very interestingly, it was found that the layer of chitosan coating (Figure 2c) significantly increased the photostability of ICG, when compared to the uncoated LDH–NH_2_–ICG nanoparticles. The photoprotection provided to ICG by chitosan-coated LDH may arise from the decreased leaching and attractions between LDH–NH_2_ surfaces and ICG molecules. Thus, the increase of absorbance may come from the decrease of aggregated forms of ICG. However, the second coating of chitosan has a decreased the residual amount compared to the primary coating because too much chitosan coating may further decrease the attractions between the ICG molecules and the LDH surfaces; therefore, the ICG molecules in the confined interlayer spaces have a random orientation to further cause the self-aggregation of the ICG dyes. These results confirm that the chitosan-coated LDH–NH_2_–ICG is beneficial for the structural stability of ICG, by clearly improving the resistance of ICG towards light degradation.

### 2.3. Cell Uptake and in Vitro Cell Imaging

**Figure 3:**
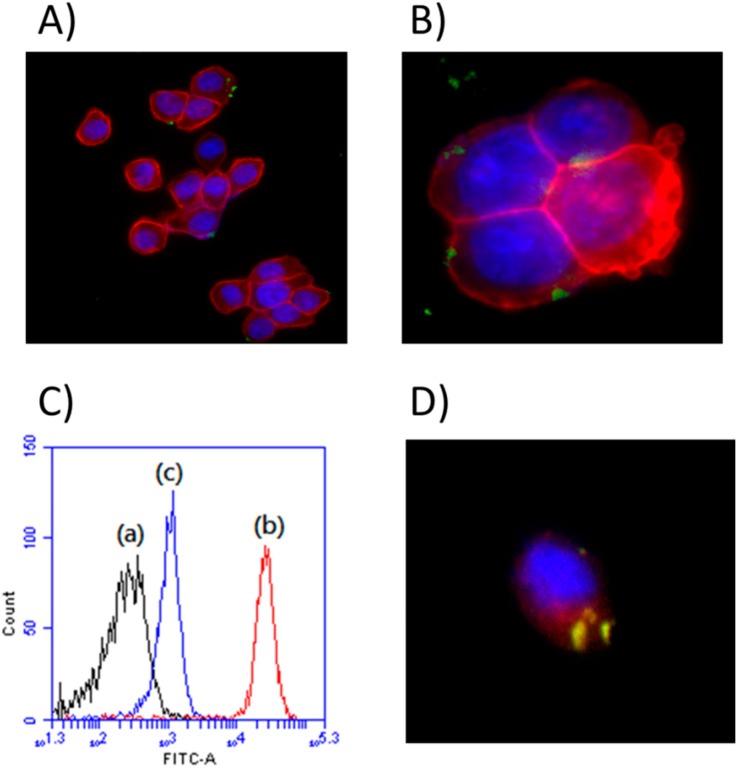
Cellular uptake imaging of LDH–NH_2_–FITC–CS nanoparticles after 4 h of incubation and captured at various magnifications, (**A**) 20× and (**B**) 60×; (**C**) Flow cytometric analyses of cell uptake of FITC conjugated LDH–CS (a) Control; (b) LDH–NH_2_–FITC–CS and (c) LDH–NH_2_–FITC–CS with Trypan blue addition; (**D**) Staining of nanoparticles (green) in the lysosomal compartment in HT-29 cells. The nuclei were stained with DAPI (blue) and the lysosomes were visualized by a lysotracker (red) (magnification, 60×).

Previous reports suggest that LDH nanoparticles are taken up by clathrin-mediated endocytosis, which progresses from early endosomes to multi-vesicular bodies/late endosomes and finally to lysosomes with a significant drop in pH [61,62]. It was thus highly desirable to understand the internalization of nanoparticles into the endosomal processing and track the uptake pattern as these processes, which plays a very key role in drug delivery design. In our study, we have used HT-29 cells treated with the fluorescent dye (Fluorescein isothiocyanate (FITC)) labelled LDH to measure the intracellular fluorescent intensity by using an Olympus fluorescence microscope. All images were obtained under identical conditions and cell nuclei were stained by 4,6-diamidino-2-phenylindole (DAPI). Fluorescent images of HT-29 cells (Figure 3A,B) exhibited significant intracellular staining, in addition to indicating accumulation of nanoparticles (light green under the dark field) around the nucleus (deep blue), emitting a strong fluorescent signal after 4 h of incubation.

The use of trypan blue as an extracellular fluorescence quenching to quantitate the intercellular fluorescent intensity was previously described. The true fluorescence of internalised FITC labelled LDH in the cells were quantified after quenching of extracellular fluorescence with trypan blue (0.4% in phosphate-buffered saline PBS) (Figure 3C(c)) which distinguishes the internalized from surface-adherent particles. In addition, the cells were incubated without FITC-labelled material (*i.e*., control) (Figure 3C(a)); the quantified fluorescence of the internalized FITC labelled LDH is shifted towards right to the control after trypan blue surface quenching, whereas the fluorescence in the cells without trypan blue treatment is relatively higher (Figure 3C(b)). This concomitantly shows the successful internalization of the chitosan-coated LDH–NH_2_–FITC.

The endocytosis pathway elucidation of LDH nanoparticles was performed using a lysotracker dye *i.e.*, a fluorescent tracer to track lysosome behaviour in internalization studies. By this approach, the lysosomal vesicles in the cells were labelled with the lysotracker dye. Therefore, we could observe the colocalization of lysoendosomal vesicle and nanoparticles in the internalization process. The excess dye was washed out, and the cells were exposed to nanoparticles. Later, the images were captured under the fluorescence microscope after 4 h of incubation period. The results revealed that the LDH nanoparticles (green fluorescence) were internalized into the lysosomes after 4 h of incubation (Figure 3D), as was evident from the co-localization of the red and green fluorescence signal. Importantly, the uptake of LDHs by cancer cells is a prerequisite for PDT treatment.

### 2.4. Determination of Singlet Oxygen Generation

**Figure 4:**
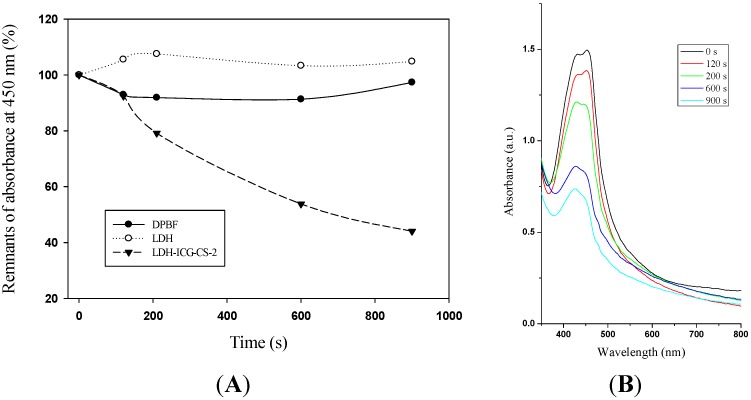
(**A**) 1,3-Diphenylisobenzofuran (DPBF) quenching of singlet oxygen generation by LDH–NH_2_–ICG–CS-2 with increasing time period of light exposure in comparison to LDH and DPBF alone; (**B**) UV–Vis curve of the DPBF quenching of singlet oxygen generation from LDH–NH_2_–ICG–CS-2 under near-infrared (NIR) excitation with increase in exposure time.

PDT of cancer cells was achieved with ROS-induced cell death from the energy transfer of excited photosensitizer to surrounding oxygen. Singlet oxygen production by ICG in the cells was confirmed using 1,3-diphenylisobenzofuran (DPBF) for singlet oxygen quencher. After light irradiation DPBF gradually decreased the absorption at the absorption maximum (~450 nm). Afterwards the LDH–NH_2_–ICG–CS-2 was exposed to NIR light (765 nm) for 15 min. As expected, the absorption of DPBF in the LDH–NH_2_–ICG–CS-2 solution showed a rapid decrease (~20%) in optical density (OD-450 nm) within 200 s of exposure time, and then declined rapidly (~60%) after 900 s of irradiation (Figure 4). This clearly demonstrates that the singlet oxygen production increased with the irradiation time in LDH–NH_2_–ICG–CS-2 samples. The results indicated that most of the LDH–NH_2_–ICG–CS-2 in the excited state might transfer their energy to surrounding oxygen upon irradiation by NIR light. To confirm that the singlet oxygen was produced by LDH–NH_2_–ICG–CS-2, control groups containing the same concentration of LDH nanoparticles and DPBF alone were evaluated under NIR light irradiation, respectively.

It was noted that the DPBF absorption in the naked LDH solution increased slightly (<2%) in the light after irradiation for 900 s, whereas a (~5%) decrease in absorption was noted in samples containing DPBF alone by continuous NIR light irradiation for 900 s. These results imply that LDH–NH_2_–ICG–CS-2 was effectively activated by the emission from ICG and resulted in singlet oxygen production. In addition, the singlet oxygen generation by LDH–ICG–CS-2 was observed with various time periods of light exposure. This indicates that the singlet oxygen generation and the respective quenching by DPBF occurred in a time-dependent manner (Figure 4B). Our experiments reveal that LDH–NH_2_–ICG–CS-2 could effectively generate singlet oxygen upon continuous NIR light excitation due to higher activity of LDH–ICG–CS-2 nanocomposites.

### 2.5. Phototherapy Assay

**Figure 5:**
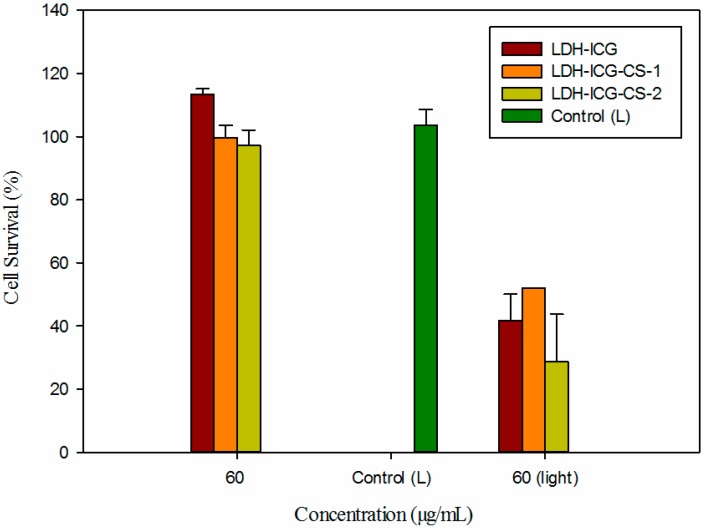
Comparison of cell viability in HT-29 cell line tested against various composite nanoparticles for different treatments: LDH–NH_2_–ICG, LDH–NH_2_–ICG–CS-1 and LDH–NH_2_–ICG–CS-2 composite nanoparticles with and without NIR irradiation. Untreated HT-29 cells were used as a control group.

Cell viability was measured as a function of phototherapy with an incubation concentration of nanocomposites at 60 μg/mL treated with LDH–NH_2_–ICG, LDH–NH_2_–ICG–CS-1 and LDH–NH_2_–ICG–CS-2 with and without light exposure. In addition, for the control experiments, the cells were incubated without nanoparticles. In the absence of NIR irradiation the cell viability of all of the nanoparticle treated samples were more or less similar to the control experiment and no cytotoxicity was observed at a concentration as high as 60 μg/mL. However, upon exposure to NIR irradiation at 60 μg/mL the cell viability of LDH–NH_2_–ICG treated cells decreased to 40% and LDH–NH_2_–ICG–CS-1 treated groups decreased to 50%. Very interestingly, the LDH–NH_2_–ICG–CS-2 nanocomposites exhibited enhanced photo cytotoxicity with cell survival reduced to 30%, which is less than the former two nanocomposites (Figure 5). The chitosan-coated LDH–NH_2_–ICG nanoparticles with better biocompatibility and stability could effectively stabilize the ICG photosensitizer and produce a high PDT effect under NIR light irradiation.

### 2.6. Apoptosis

The primary site of photodamage accords with the intracellular localization of the photosensitizers, due to the short life span and limited diffusion capability of photogenerated reactive oxygen species into the biological systems [63,64]. Thus, primary molecular targets of PDT should reside within a short distance from the photosensitive molecule. In general, photosensitizers may localize at the cell mitochondria, endoplasmic reticulum and the Golgi apparatus to cause cell apoptosis. Furthermore, the photosensitizers may target the cell membranes to trigger ROS and make the cells vulnerable to necrosis [65]. That internalized ICG-loaded LDH nanoparticles could target mitochondria and subsequently affect the mitochondrial membrane potential, which directly reflects cell survival, was verified. The mitochondrial membrane potential (MMP) was investigated, such as the depolarization of the MMP before and after PDT with LDH–NH_2_–ICG. The determination of membrane potential was measured using a JC-1 (tetraethylbenzimidazolylcarbocyanine iodide) assay, since it is a hydrophobic dye with a positive charge that can penetrate inner mitochondria membranes and change fluorescence from green to red as the membrane potential increases. Thus, the hyper polarization state of mitochondria membrane in the healthy cells displayed intense red fluorescence of J-aggregates. On the other hand, the apoptotic cells were in a depolarized state, where JC-1 remains in the monomeric form, and thus a higher green fluorescence was observed.

**Figure 6:**
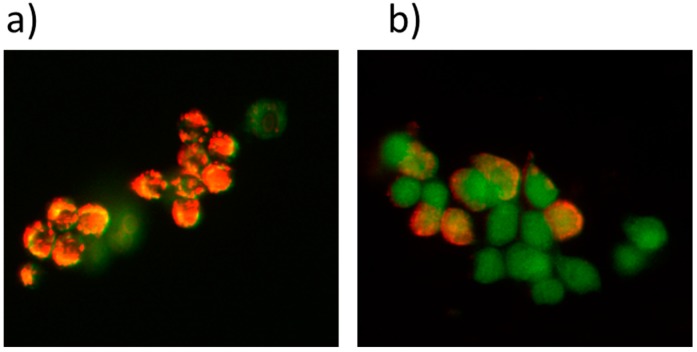
Effect of LDH nanoparticles on mitochondrial membrane potential in HT-29 cells. (**a**) Control cells with LDH–ICG nanoparticles show most cells had a strong J-aggregation (red) in the absence of light irradiation; (**b**) drug-loaded LDH nanoparticles show a majority of these cells emitted green fluorescence, due to low mitochondrial membrane potential after treatment with LDH–NH_2_–ICG under light irradiation (magnification of both images at 40×).

Figure 6 shows that no detectable loss of membrane potential was found in cells treated with ICG loaded nanoparticles in the absence of light irradiation. However, LDH–ICG had a great effect on the loss of MMP upon light irradiation. A majority of cells treated with ICG-loaded LDH nanoparticles exhibited a green fluorescence (<90%) after light irradiation, indicating the disruption of membrane potential. Furthermore, further cell death may come from the dissipation of membrane potential which may result in the decrease of mitochondrial ATP levels. The results showed that the LDH–NH_2_–ICG nanoparticles serve as an effective ICG nanocarrier system for killing cancer cells under light irradiation (Figure 6).

**Figure 7:**
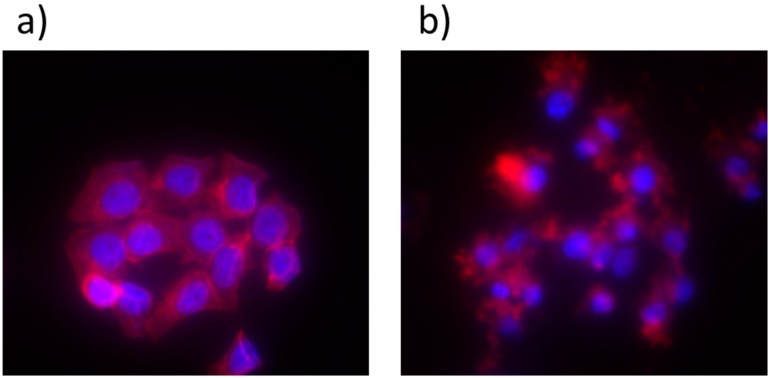
PDT-induced nuclear morphology change in HT-29 cell line. (**a**) BD (Becton Dickinson’s pathway) images of nuclear morphology in control HT-29 cell after staining with DAPI upon treatment with nanoparticles without phototherapy; (**b**) Images of nucleus that represent the typical apoptotic morphological changes such as nuclear condensation, upon nanoparticle treatment in HT-29 cell line with phototherapy (magnification of both images at 40×). Red and purple stain represents the cytoskeleton and nucleus of the cell respectively.

**Figure 8:**
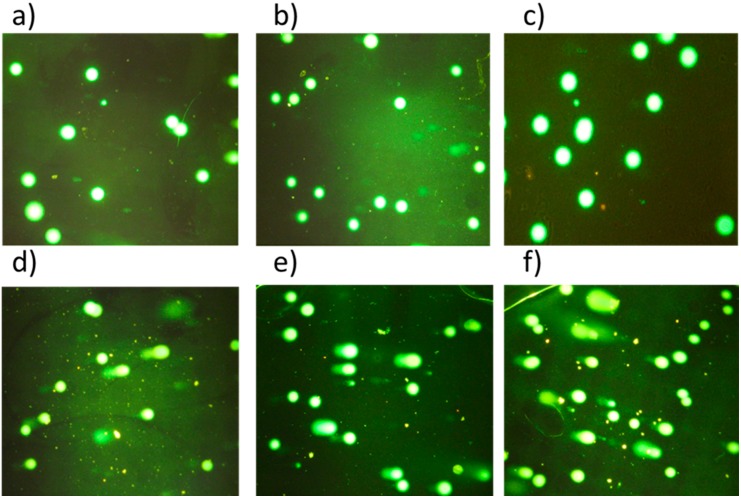
Microphotographs of a comet image in the HT-29 cells: (**a**) Untreated control without light (undamaged cell); (**b**) HT-29 cells treated with LDH; (**c**) Untreated control with light; (**d**) Cells treated with LDH–NH_2_–ICG with light; (**e**) LDH–NH_2_–ICG–CS-1 and (**f**) LDH–NH_2_–ICG–CS-2. Nuclei were stained with SYBR^®^ Green nucleic acid gel stain (magnification of all of the images captured at 40×).

The mechanism of PDT induced cytotoxicity was also evaluated by investigating the changes of cell morphology after PDT in HT-29 cells through staining with 4,6-diamidino-2-phenylindole (DAPI) for 4 h. The nuclear changes and chromatin condensation were seen after light irradiation (Figure 7). The phenomenon indicated the changes of the chromatin morphology from the apoptotic cells. Further, DNA fragmentation by comet assay is a typical sign of cell apoptosis after PDT in HT-29 cells (Figure 8).

DNA damage was further evaluated by comet assay. The comet assay results showed significant DNA damage of HT-29 cell line (as indicated from tail moment) after PDT using LDH–NH_2_–ICG (Figure 8d), LDH–NH_2_–ICG–CS-1 (Figure 8e) and LDH–NH_2_–ICG–CS-2 (Figure 8f). The effects of various treated nanoparticles showed no damage in the controls with (Figure 8c) and without light irradiation (Figure 8a) as well as LDH-treated cells (Figure 8b), whereas cells treated with LDH–NH_2_–ICG with light showed slighter damage and chitosan-coated samples for primary coat and secondary coat have more DNA fragmentation than former treatments. The mechanistic issues of DNA damage induced by PDT are not yet clear. Previous studies showed that PDT can cause DNA base oxidation, DNA cross-linking, protein heat shock and exchange of sister chromatids [66,67,68]. PDT may also involve the generation of various free radicals and reactive oxygen (hydroxyl radicals, superoxide and singlet oxygen). The singlet oxygen showed very near distance and short life-time. Thus, it showed little possibility for the trigger DNA damage through singlet oxygen production, unless the ROS are generated very close to the cell nucleus. On the other hand, reactive oxygen species (ROS) is capable of inducing oxidative DNA damage and was believed to be a major cause for DNA damage [69].

Recent studies confirmed that PDT is capable of damaging cytoplasmic proteins and mitochondria leading to the arrest of the cell cycle to cause the cell apoptosis [70]. PDT using LDH–NH_2_–ICG has been shown to have interference with mitochondrial membrane potential along with considerable DNA damage, which is in a good correlation with the report from Miller *et al.* confirming the PDT effect of synthesized nanoconjugates [70]. In the present study, it was also obvious that the extent of DNA damage as represented by the tail moment was higher in the cells treated with LDH–NH_2_–ICG–CS followed by laser than the cells treated with LDH–NH_2_–ICG followed by laser, and this could be an indication that using chitosan coating in PDT can enhance the singlet oxygen to damage DNA. Therefore, the use of LDH–NH_2_–ICG–CS nanocomposites for PDT takes advantages of increasing the photosensitizer stability and DNA damage.

**Figure 9:**
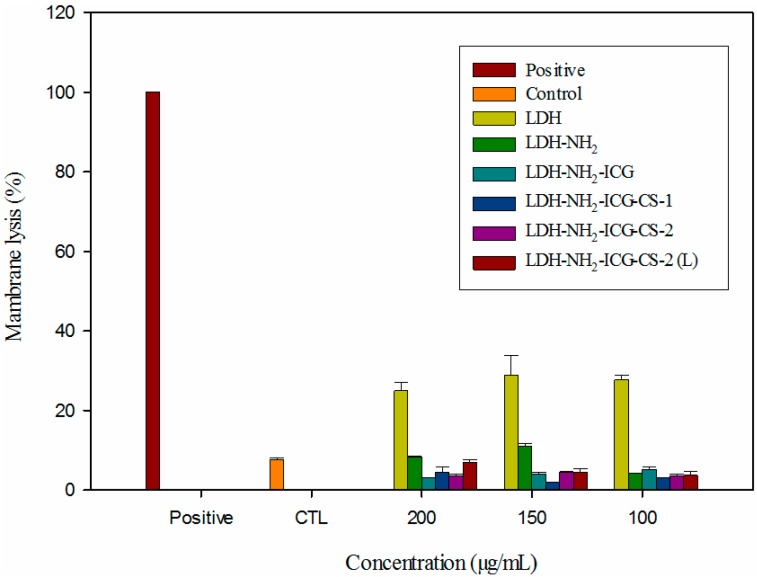
Lactate dehydrogenase assay in the HT-29 cell line tested against various composite nanoparticle (LDH, LDH–NH_2_, LDH–NH_2_–ICG, LDH–NH_2_–ICG–CS-1, LDH–NH_2_–ICG–CS-2 and LDH–NH_2_–ICG–CS-2 (L)) for different treatments: with and without NIR irradiation. Untreated HT-29 cells were used as a control group, and compared with the positive control (triton X-100).

The lactate dehydrogenase assay quantitatively measures the activity of the enzyme (lactate dehydrogenase) that is released upon cell membrane damage. In this experiment, the maximal release was obtained by the treatment of control cells (untreated cells) with 0.1% triton X-100 (positive control) for 10 min at room temperature. Compared with the control experiment (CTL), treatment with naked LDH demonstrated a considerable amount of lactate dehydrogenase release (calculated using Equations (1) and (2) as specified in experimental section), which may arise from the strong interactions between positively charged LDH interlayers with the negatively charged biological membranes. The surface-functionalized LDH nanoparticles have considerably decreased release of lactate dehydrogenase, even at doses as high as 200 μg/mL, when compared to the non-functionalized nanoparticles (100 μg/mL) suggested that the functionalization of nanoparticles could significantly reduce their cytotoxic effects of nanoparticles by decreasing the affinity of nanoparticles towards the cells. Compared with the control experiment (CTL), the LDH–NH_2_, LDH–NH_2_–ICG, LDH–NH_2_–ICG–CS-1, and LDH–NH_2_–ICG–CS-2 samples did not show a profound increase in the release of lactate dehydrogenase after treatment with various concentrations (100, 150 and 200 μg/mL) for 24 h, which could be attributed to the decreased surface charge with enhanced biocompatibility provided by the chitosan matrix. The release of lactate dehydrogenase was less than the CTL cells when treated with LDH–NH_2_–ICG-2 complexes in the presence of light at a various concentrations (Figure 9). It is concluded from the obtained results that both the drug-conjugated and chitosan-coated LDH nanoparticles are highly biocompatible, retaining the membrane integrity with no cell necrosis observed. A majority of anti-cancer drugs induce cell death either by apoptotic or necrotic pathways. Induction of cell death by employing the apoptotic pathway offers many benefits over the necrotic pathway, which involves the breakage of cell membranes releasing the cytokines that can trigger inflammatory responses, which in turn can result in a poor prognosis.

**Figure 10:**
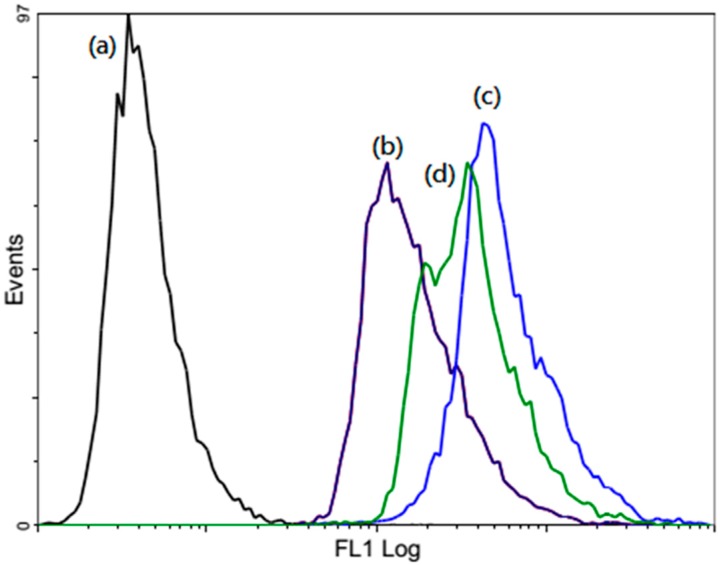
Detection of ROS using 2′,7′-dichlorodihydrofluorescin diacetate (DCFH-DA) HT-29 Cells were incubated (a) without nanoparticle (control); (b) LDHs–NH_2_–ICG; (c) LDHs–NH_2_–ICG–CS-1; (d) LDHs–NH_2_–ICG–CS-2 (100 µg/mL). DCF fluorescence was detected by flow cytometry.

The intracellular ROS generated by the nanocomposites were determined by using a fluorescent dye 2′,7′-dichlorodihydrofluorescein diacetate (DCFH-DA), which may undergo oxidation by intracellular ROS to produce a fluorescent 2′,7′-dichlorofluorescein (DCF) molecule. Compared to the control group (Figure 10a), the entire nanoparticle treated groups (LDH–NH_2_–ICG (Figure 10b), LDH–NH_2_–ICG–CS-1 (Figure 10c) and LDH–NH_2_–ICG–CS-2 (Figure 10d) induced high generation of ROS. The ROS signal induced by chitosan coated nanoparticles was remarkably higher than the ROS generated by LDH–NH_2_–ICG alone in accordance with the PDT effect (Figure 5) and singlet oxygen production (Figure 4), which suggests that the coating of chitosan imparts highly beneficial properties to the nanocomposites. We can summarize the results of the photodynamic therapy as follows: the increased levels of ROS (singlet oxygen) after light irradiation may decrease the mitochondria membrane potential to further increase the mitochondria membrane permeability. Subsequently the loss of electrostatic attractions between the cytochrome *c* and intermembranes may cause the release of cytochrome c into the cytosol to trigger the activation of the apoptosis protease and production of the apoptotic body (apoptosome). Thus, the production of ROS from the PDT may accumulate high levels of singlet oxygen to cause DNA breaking and fragmentation.

## 3. Experimental Section

### 3.1. Materials

Magnesium nitrate hexahydrate [Mg(NO_3_)_2_·6H_2_O], aluminium nitrate nonahydrate [Al(NO_3_)_3_·9H_2_O], indocyanine green (ICG), 2′,7′-dichlorodihydrofluorescein diacetate (DCFH-DA), 4,6-diamidino-2-phenylindole (DAPI), 3-(4,5-dimethylthiazol-2-yl)-2,5-diphenyltetrazolium bromide (MTT), glutaraldehyde (CH_2_(CH_2_CHO)_2_), chitosan (low molecular weight), potassium phosphate monobasic (KH_2_PO_4_), sodium phosphate dibasic (Na_2_HPO_4_), potassium phosphate dibasic (K_2_HPO_4_) and potassium bromide (KBr) (FT-IR grade) were purchased from Sigma-Aldrich (St. Louis, MO, USA). 3-Aminopropyl-trimethoxysilane (APTS) was obtained from Gelest (Morrisville, PA, USA). NaOH and DMSO were purchased from Acros Organics Ltd. (Loughborough, UK).

### 3.2. Characterization and Instruments

Fourier transform infrared (FT-IR) spectral measurements were taken using Bruker Alpha spectrometer (Billerica, MA, USA. TEM images were captured on a Hitachi H-7100 (Hitachi High Technologies Corporation, Tokyo, Japan) operating at 100 kV. Aqueous LDH sample was deposited on carbon coated copper (Cu) grids and dried at room temperature. Thermogravimetric analysis-differential thermal analysis (TGA-DTA) using TGA Q50 V20, 13 Build 39 (Universal V4.5A TA Instruments, New Castle, PA, USA). Powder X-ray diffraction (PXRD) spectra were recorded using a X-ray diffractometer (XRD D8 Advanced, Bruker, Karlsruhe, Germany) at a diffraction angle (2θ) from 2° to 70° with a scanning speed of 3°·min^−1^. Centrifugation during the nanomaterial synthesis and cell culturing process was performed at an appropriate temperature and rotations per minute (rpm) using Hermle Z 36 HK (HERMLE Labortechnik GmbH, Wehingen, Germany) instruments and Kubota KN-70 (Kubota Corporation, Tokyo, Japan) respectively. Ultraviolet–visible (UV–Vis) spectroscopic absorbance was documented on a Genequant-1300 series spectrophotometer (GE Healthcare Biosciences, Pittsburgh, PA, USA). Fluorescence images were captured on an Olympus microscope hybridized with Nikon CCD camera apparatus (Nikon instruments, Palo Alto, CA, USA) and with BD (Becton Dickinson) pathway (BD biosciences, San Jose, CA, USA). Fluorescence intensity and MTT (3-(4,5-dimethylthiazol-2-yl)-2,5-diphenyltetrazolium bromide) absorbance were recorded using EnSpire Multi-label Plate Reader (Perkin Elmer Inc., Santa Clara, CA, USA). The flow cytometric quantification of ROS was performed using a Cytomics FC-500 (Beckmann Coulter Inc., New York, NY, USA) equipped with a FSC (Forward-scattered Light) detection system and argon laser lamp.

### 3.3. Synthesis of LDH Nanoparticles

LDH nanocontainers were prepared using a co-precipitation method as reported previously [19,71,72,73,74]. 0.769 g of magnesium nitrate hexahydrate [Mg(NO_3_)_2_·6H_2_O] and 0.375 g of aluminium nitrate nonahydrate [Al(NO_3_)_3_·9H_2_O] were dissolved in 10 mL dd-H_2_O in a vacuum-packed container and quickly added to 40 mL of NaOH solution (0.15 M) by stirring at room temperature under high nitrogen purge for 10 min. Atmospheric CO_2_ contamination was prevented successfully using decarbonated dd-H_2_O used in all of the preparations. The nanoparticles were washed eventually with 40 mL of dd-H_2_O and centrifuged at 10,000 rpm for 10 min to collect the nanoparticles. The as-synthesized products were further suspended in 50 mL dd-H_2_O and the solution underwent hydrothermal treatment (100 °C for 16 h) using Teflon-lined autoclave. The nanoparticles were subsequently collected by centrifuging at 10,000 rpm for 10 min, washing and then stored in ethanol to prevent contamination.

### 3.4. Synthesis of LDHs–NH_2_

Surface amine modification using 3-aminopropyl-trimethoxysilane (APTS) molecules on the LDH surfaces was attained efficiently using toluene as the reaction solvent, and the condensation of APTS molecules and LDH surfaces was achieved well under a high reflux temperature [19,71] and the synthesis was monitored. 0.2 g of the as-synthesized LDHs nanoparticles were placed in 30 mL toluene and stirred for 30 min; later, 1.0 mL of APTS was added to the resultant mixture under nitrogen atmosphere. The reaction was carried out at 100 °C for 24 h, followed by particle collection by centrifugation (10,000 rpm, 10 min, at 4 °C) and washed thoroughly with acetone and ethanol. The nanoparticles were dispersed in ethanol for further modification.

### 3.5. Synthesis of LDHs–NH_2_–ICG

Spacer glutaraldehyde was conjugated at first on LDHs–NH_2_ surfaces before ICG loading (LDHs–NH_2_–GA (glutaraldehyde) samples) and as follows: LDHs–NH_2_ (15 mg) was suspended in anhydrous methanol (0.8 mL) and the nanoparticles were further treated with glutaraldehyde (0.4 mL and stirred for 6 h) at room temperature. Later on, the nanoparticles were collected by centrifugation at 10,000 rpm for 10 min and washed subsequently with methanol once. Since the ICG molecule was highly negatively charged, the framework of LDHs can provide a strong electrostatic attraction and can be helpful to increase the loading amounts of ICG molecules in LDHs–NH_2_–GA sample layers. The further intercalation of ICG molecules in LDHs–NH_2_–GA surfaces were as follows: at the very beginning, 1 mg of ICG was added in 1 mL methanol and then mixed with the LDHs–NH_2_–GA samples (15 mg). The mixture was stirred at room temperature for 1 h and the products (LDHs–NH_2_–ICG) were washed with methanol twice by centrifuging at 10,000 rpm for 10 min.

### 3.6. Synthesis of Chitosan-Coated LDHs–NH_2_–ICG

The polymer was coated around the LDH surface using the amine linker as well as glutaraldehyde spacer and the primary coat (LDHs–NH_2_–ICG–CS-1) sample was prepared as follows: LDHs–NH_2_–ICG (15 mg) samples were added to 1 mL of chitosan solution (6 mg/mL in 1% AcOH) by shaking the mixture in the orbital shaker at 37 °C and 200 rpm for 3 h. Thus, the aldehyde groups in the LDHs–NH_2_–ICG surfaces were able to react with the amino groups of the chitosan molecules by the glutaraldehyde modification. The final coated products were collected by centrifugation (10,000 rpm for 10 min) and then washed with dd-H_2_O twice. Moreover, the synthesis of the secondary sample (LDHs–NH_2_–ICG–CS-2) was achieved by repeating the above reaction using LDHs–NH_2_–ICG–CS-1 samples.

### 3.7. Synthesis of LDH–NH_2_–FITC–CS (Fluorescein Isothiocyanate–Chitosan)

The FITC–CS complex was prepared and then conjugated to the nanoparticle, which served as a tracer. The fluorescence tag was conjugated to chitosan by adding 5 mL of FITC in methanol (2.0 mg/mL) to 10 mL of chitosan solution (10 mg/mL in 0.1% AcOH in water) in the dark at ambient temperature maintained at pH-8.0 for 3 h. Later on, the polymer was precipitated in 0.2 M NaOH and the precipitate was pelleted at 12,000 for 10 min and washed once with ethanol:water (70:30 *v*/*v*) and this was repeated until no fluorescence was detected in the supernatant. Further, tagging was done as follows: surface amination was performed to the as-synthesized LDH (0.2 g) using APTS (see Section 3.4.) and later, FITC–CS was dissolved in 10 mL of dry methanol and added to 100 mg of nanoparticles (LDH–NH_2_), re-suspended and stirred for 24 h in the dark at room temperature.

### 3.8. Photostability Assay

Dye stability is improved when encumbered in the nanocarrier; the stability was tested after irradiation with specific light for a specified time period of exposure and the method as follows. One mg of each nanoparticle sample (LDH–NH_2_–ICG, LDH–NH_2_–ICG–CS-1, LDH–NH_2_–ICG–CS-2) stored in ethanol was centrifuged at 10,000 rpm for 10 min. The nanoparticles were resuspended in 1 mL of double distilled water and then placed in an orbital shaker incubator. In addition, 1 mg of ICG dye alone was taken separately in 1 mL of double distilled water and processed similarly for comparison. The samples were exposed to light irradiation only for 3 min and after three hours the sample’s absorbance was read using a UV–Vis spectrophotometer at 765 nm. The changes were recorded before and after light irradiation. The residual amount was calculated using the formula: (OD (optical density) after irradiation) × 100/(OD before irradiation).

### 3.9. Cellular Uptake and Imaging by Confocal Microscopy

Cell uptake was performed by seeding 3 × 10^3^ HT-29 cells/well in the 96-well plate and culturing for 24 h. LDH–NH_2_–FITC–CS samples were added (10 μg/mL) and incubated for 24 h. Treated cells were washed several times with PBS and fixed with formaldehyde (3.7%) for 10 min at room temperature. The cells were washed thrice with PBS and incubated with 0.1% triton X-100 and then 3% bovine serum albumin (BSA) in PBS for 5 and 30 min, respectively. Rhodamine phalloidin was used for staining the filamentous actin skeleton at room temperature for 20 min. The nucleus was stained with DAPI (2 µg/mL in H_2_O) for 5 min. The samples were observed using confocal microscopy.

### 3.10. Lysosome Staining

HT-29 cells were seeded in 96-well plate and cultured for 24 h. LDH–NH_2_–FITC–CS (10 μg/mL) samples were added to the cultured cells and incubated for another 24 h. Further, 50 nM LysoTracker Red was added to the cells and incubated for 30 min. The cells were washed with PBS to remove excess stain and visualized under a fluorescent microscope.

### 3.11. In Vitro Determination of Singlet Oxygen Production by 1,3-Diphenylisobenzofuran (DPBF)

Singlet oxygen production was determined by DPBF bleaching method [65]. 1,3-Diphenylisobenzofuran (DPBF), a sensitive probe was used to detect singlet oxygen production during the nanoparticle photosensitization. Upon oxidative degradation by ^1^O_2_, the fluorescent DPBF changes to non-fluorescent *O*-dibenzoylbenzene, so that the reducing rate of DPBF fluorescence in sample solution, which is proportional to the ^1^O_2_ production, can be used to measure the relative yield of ^1^O_2_. 1 mg of each nanoparticle sample (LDH and LDH–NH_2_–ICG–CS-2) was pelleted by centrifugation (12,000 rpm, 10 min) and 1 mL of double distilled water is added to each sample. DPBF (8 mM) was added into each nanoparticle sample individually and irradiated with light for the respective time intervals. The particles were centrifuged (12,000 rpm, 10 min) and UV–Vis readings were recorded.

### 3.12. Photodynamic Effect Measurements

Cell culture: HT-29 (colon cell line) cells were cultured in an Roswell Park Memorial Institute (RPMI)-1640 medium supplemented with 10% (*v*/*v*) fetal bovine serum (FBS) and penicillin (100 units·mL^−1^)/streptomycin (100 mg mL^−1^). Cultures were maintained in a humidified incubator at 37 °C in 5% CO_2_.

To evaluate the influence of the ICG-based PDT on cell growth, the investigated cells were irradiated using a diode laser (MC LASER, therapy laser processor PMC-018, Katowice, Proland). All irradiations were performed at 830 nm and at a fluence rate of 100 or 200 mW/cm^2^ with the doses from 30 to 100 J/cm^2^ in the presence of the photosensitizer (100 µM). After irradiation the cells were incubated in the culture medium for 24 h. Surviving cells (viable cell count) were monitored by counting the number of untreated and PDT treated cells using MTT assay [7].

### 3.13. Measurements of the Mitochondrial Membrane Potential

The cationic fluorescent probe JC-1 exists as a monomer (green) at low membrane potential, and at higher potentials, JC-1 forms red fluorescent aggregates [75]. HT-29 cells (1 × 10^4^ cells/mL), were seeded in 96-well plates, followed by incubation with LDH–ICG–CS-2 for 4 h, and exposed to radiation for 5 min and incubated for 24 h. Later, cells were resuspended in 500 µL of PBS (pH7.2) containing 2 mM of JC-1 and incubated at 37 °C for 30 min and observed under a fluorescent microscope.

### 3.14. Comet Assay

The comet assay was performed according to the manufacturer’s (Trevigen’s Comet Assay^®^ kit (Trevigen Inc., Helgerman court, Gaithersburg, MD, USA) instructions. HT-29 cells were seeded at a density of 2 × 10^5^ cells/well in a 6-well plate and LDH–NH_2_–ICG, LDH–NH_2_–ICG–CS-1, LDH–NH_2_–ICG–CS-2 were added. Four hours after incubation, the light was irradiated to a specified time period and then the plates were incubated for 20 h. The harvested cells were pooled in a 1% low melting point agarose 1:10 (*v*/*v*). The electrophoresis was performed in accordance with the specifications (20 min at 21 Volts and 350 mA) provided. After washing with water and ethanol to reanneal the DNA, the smear was stained with SYBR Green I (Molecular Probes, Eugene, OR, USA) (1 mg/mL). Micrographs were captured using Olympus fluorescence image analysis.

### 3.15. Flow Cytometric Detections

Intracellular ROS (reactive oxygen species) levels were measured using the dichlorodihydrofluorescein diacetate (DCFH-DA) assay. HT-29 cells (2 × 10^5^ cells/well), were seeded in a 6-well plates, followed by incubation with LDH–NH_2_–ICG, LDH–NH_2_–ICG–CS-1, LDH–NH_2_–ICG–CS-2 nanoparticles for 24 h. Cells were stained for cellular ROS by incubation with 20 μM DCFH-DA (Invitrogen, Eugene, OR, USA) for 30 min at 37 °C. DCFH-DA after entry into cell becomes oxidized by radicals such as hydroxyl, peroxyl, alkoxyl, nitrate and carbonate to a fluorescent molecule (excitation 485 nm, emission 530 nm). The cells were scraped and the supernatants were centrifuged and cells were exposed to radiation for 10 min. Fluorescence levels of the ROS levels were conducted approximately 90 min post-irradiation using flow cytometry.

Quantification of internalized FITC-labelled LDH in HT-29 cells by flow cytometry was measured following the procedure with slight modifications [76]. Cells were seeded at the density of 2 × 10^5^ cells/well in a 6-well plates with 2 mL of RPMI-1640 medium supplemented with 10% (*v*/*v*) FBS. After incubation for 24 h, the medium was replaced by 2 mL of FITC labeled material suspensions (50 μg·mL^−1^) in the serum-free medium for 4 h. The cells were washed with PBS, harvested by trypsinization and after centrifugation, re-suspended in 0.4% trypan blue in PBS solution for surface quenching and analyzed by flow cytometry. Trypan blue was used to distinguish the true fluorescence generated by the endocytosed LDH–NH_2_–FITC–CS from the auto fluorescence of cells and compared with the threshold fluorescence intensity of the cells incubated without FITC-labeled material.

### 3.16. Lactate Dehydrogenase Assay

Cell proliferation by LDH release method was performed using the TOX-7 LDH based *in vitro* toxicology assay kit (Sigma Corporation Ltd., St. Louis, MO, USA). 1 × 10^5^ cells/well were seeded in a 12-well plates and incubated for 24 h. The cultured cells were treated with LDH, LDH–NH_2_, LDH–NH_2_–ICG, LDH–NH_2_–ICG–CS-1, LDH–NH_2_–ICG–CS-2 at different concentrations (100, 150 and 200 μg/mL) and further incubated for 24 h. In addition, the cells incubated with LDH–ICG–CS-2 particles were exposed to light after 4 h of incubation to allow particle uptake and exposed to light radiation for 5 min and allowed to incubate for 20 h. 0.1% triton X-100 treated cells were used as a positive control and the supernatants were collected after 1 h of incubation by centrifuging the suspension at 250× *g* for 4 min. LDH reduces NAD^+^, which converts a tetrazolium dye to a coloured formazan derivative, which is detectable spectrophotometrically at a wavelength of 490 nm and subtract a background measured at 690 nm and calculated using the Equation (1) given below. % cell lysis related to LDH release was obtained as indicator using Equation (2) by comparing the control and treatment conditions using one-way ANOVA (SigmaPlot Systat Software (SPSS) (Systat Software Inc., San Jose, CA, USA)) and Bonferroni post-hoc analyses. Each experiment was performed in triplicate and repeated three times.

(Absorbance (Abs) 490 nm − Abs 690 nm − Abs blank) = Abs Final(1)

% Cell lysis = final Abs of drug treatment/final Abs of triton X-100 treatment(2)

## 4. Conclusions

In summary, we have developed organic-inorganic hybrid nanocomposites to functionalize chitosan over LDH surfaces, making it highly biocompatible and multifunctional. These particles may serve as a delivery system for *in vivo* imaging and photodynamic therapy. A FDA approved NIR fluorescent dye, ICG, with photodynamic properties was intercalated into amine modified Mg–Al–LDH interlayers by the ion-exchange approach. An efficient positively charged polymer (chitosan) coating was achieved by the cross linkage of surface amine groups of LDH nanoparticles with amino groups of chitosan by using glutaraldehyde as a cross linking agent. The LDH–ICG–chitosan nanocomposites showed high photo-toxicity of PDT because the photosensitizers were well protected against photo and thermal degradations. Due to the deep tissue penetration of NIR light, LDH–ICG–CS-2 has great potential for *in vivo* PDT of cancer.

## Supplementary Materials

Supplementary materials can be found at http://www.mdpi.com/1422-0067/16/09/20943/s1.
