# Supplementary material for: Synthesis and Characterization of Chitosan-Coated Near-Infrared (NIR) Layered Double Hydroxide-Indocyanine Green Nanocomposites for Potential Applications in Photodynamic Therapy
Source: Int J Mol Sci. 2015 Sep 1;16(9):20943–68. doi: 10.3390/ijms160920943 (PMC4611849; doi:10.3390/ijms160920943)
Supplement: Supplementary file 1 [file ijms-16-20943-s001.pdf]

Supplementary Information

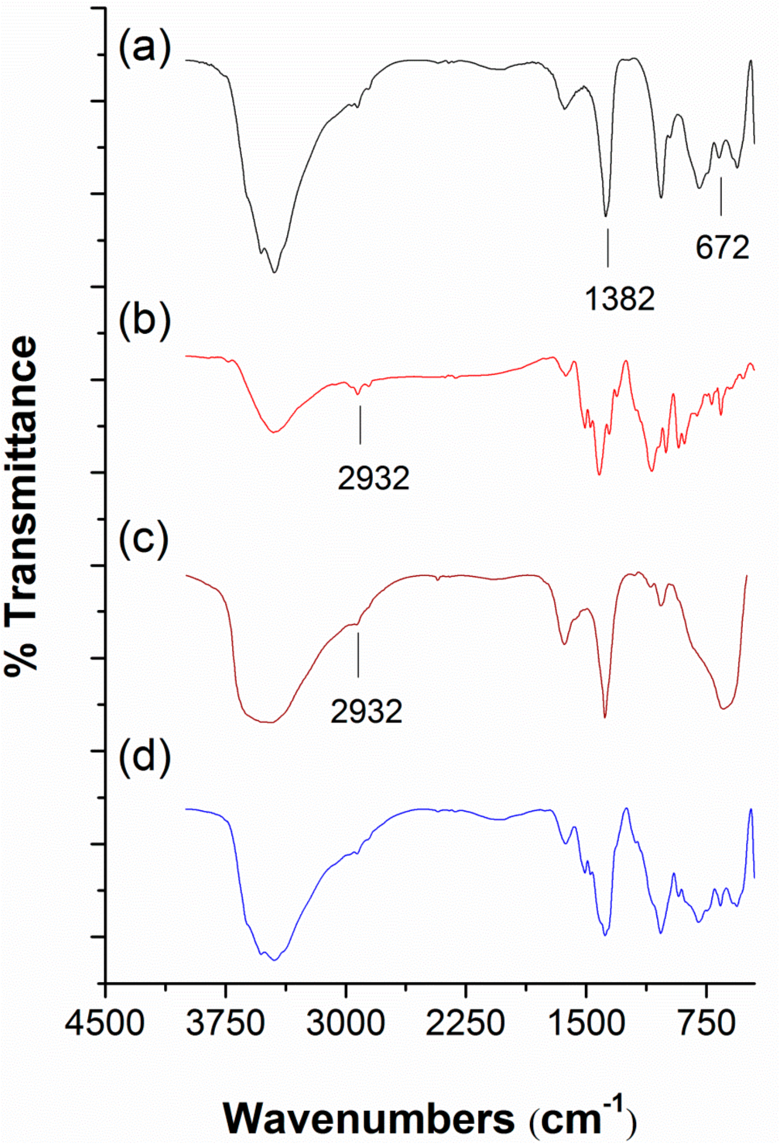

Figure S1. FT-IR spectra of (a) LDH; (b) ICG; (c) LDHs–NH_2–ICG and (d) physical mixture of LDHs–NH_2 and ICG.

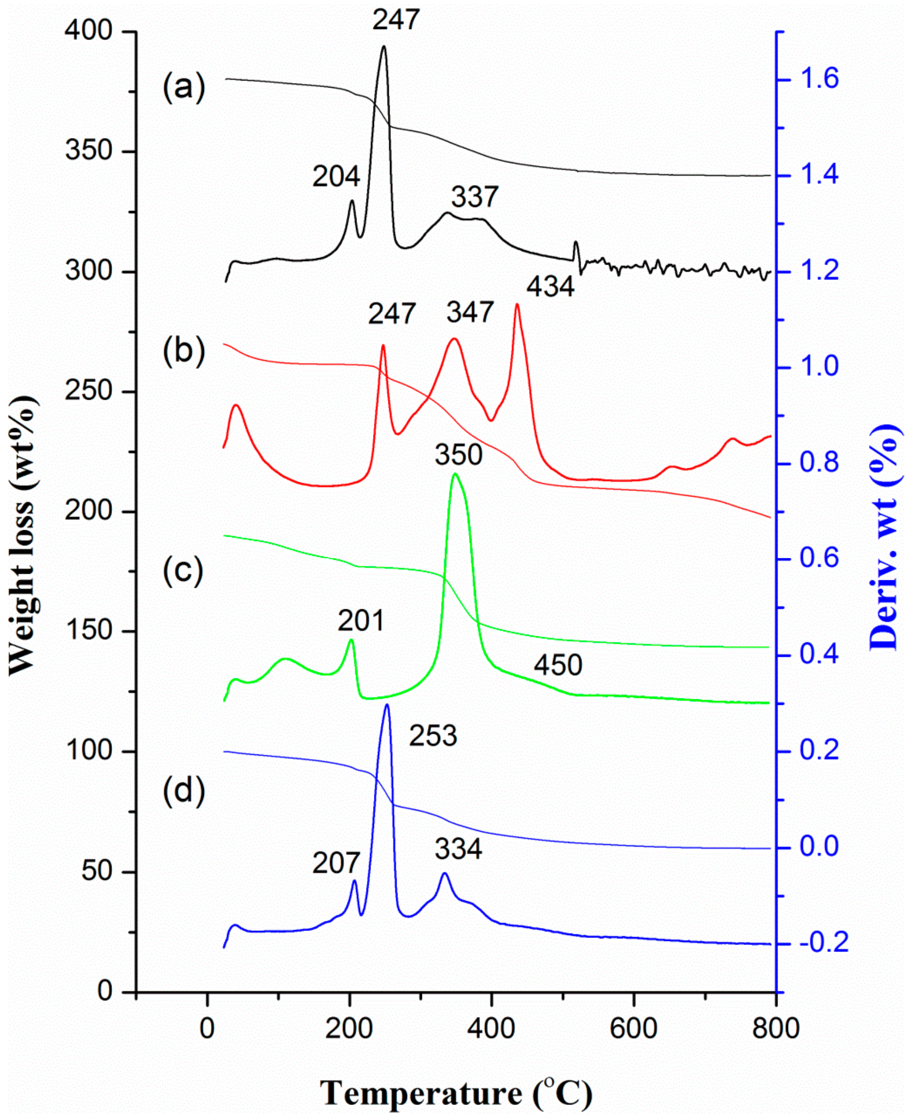

Figure S2. TGA curves of (a) LDH; (b) ICG; (c) LDHs–NH_2–ICG and (d) physical mixture of LDHs–NH_2 and ICG.
